# Cognitive and Behavioral Predictors of Quit Attempts and Biochemically-Validated Abstinence During Pregnancy

**DOI:** 10.1093/ntr/ntw242

**Published:** 2017-04-11

**Authors:** Joanne L. Emery, Stephen Sutton, Felix Naughton

**Affiliations:** 1 Behavioural Science Group, Institute of Public Health, University of Cambridge, Cambridge, UK

## Abstract

**Introduction:**

Initiating a quit attempt and achieving abstinence are distinct behaviors that have distinct correlates in general smokers. Studies predicting prenatal smoking have not addressed this.

**Methods:**

Pregnant smokers (*N* = 207), recruited to a cessation intervention trial, were used as an observational cohort. Women completed measures at baseline and 12-week follow-up (mid-late pregnancy). Outcomes were having made at least one quit attempt since baseline, and cotinine-validated 7-day abstinence at follow-up in attempters. Baseline predictors included demographics (age, deprivation, partner’s smoking), smoking behaviors (nicotine dependence, quit attempt history, previous prenatal smoking), and smoking beliefs (self-efficacy, determination, intention to quit, nonsmoker identity, social support, pregnancy-outcome beliefs). For each outcome, variables reaching *p* < .1 in logistic regression analyses were entered into a multivariate model controlling for trial arm. A complete case analysis was undertaken, with missing data assumptions tested in sensitivity analyses.

**Results:**

One hundred seventy-five women (85%) completed follow-up. Intention and determination to quit (*p* < .001), self-efficacy, nonsmoker identity, and not having previously smoked in pregnancy (*p* < .05) were univariate predictors of making a quit attempt, with stronger intention to quit the only independent predictor (multivariate odds ratio [*OR*] = 2.41, 95% confidence interval [CI] 1.19–4.87). Only nicotine dependence predicted validated abstinence among those who made a quit attempt (multivariate *OR* = 0.25, 95% CI 0.10–0.60).

**Conclusions:**

Initiating a quit attempt and achieving abstinence during pregnancy were found to have different correlates. For women yet to make a quit attempt in their pregnancy, smoking beliefs may be important intervention targets, but once they are engaged in quitting, nicotine dependence appears of prime importance.

**Implications:**

This study suggests that cognitive, particularly motivational, variables predict whether pregnant smokers will make a quit attempt, but they do not predict successful abstinence in those who attempt to quit, where nicotine dependence dominates. Interventions should facilitate quit attempts by targeting motivational variables among pregnant women who continue to smoke, but should focus on managing withdrawal once a woman initiates a quit attempt.

## Introduction

Smoking in pregnancy is associated with a wide range of adverse birth and infant health outcomes.^1,2^ It is the leading, preventable cause of miscarriage,^3^ stillbirth,^2,4^ and prematurity.^5^ In the United Kingdom, around 11% pregnant women are estimated to smoke at the time of delivery,^6^ but rates rise considerably with increasing social deprivation.^6,7^ Children born to smokers are also at an increased risk of becoming smokers themselves.^8^ Thus prenatal smoking exacerbates health inequalities, and reducing it remains a public health priority.

Up to half of pregnant smokers are estimated to quit spontaneously upon discovering their pregnancy.^9–11^ Among the remainder, the majority wish to quit,^12^ but few are successful despite repeated attempts or interventions,^13–15^ and many pregnant quitters relapse after giving birth.^11,16^ A wide range of factors have been shown to predict successful quitting in pregnancy, including demographic, behavioral, and cognitive variables.^10^ Demographic factors that consistently predict quit success in pregnancy are not having a partner who smokes,^10^ primiparity,^10,17,18^ and, less consistently, higher education,^10,18–21^ and higher age.^10,18,19^ Of behavioral/smoking-related factors, measures of nicotine dependence are consistent negative predictors of cessation.^10,20^ Fewer cognitive variables have been investigated as potential predictors, but self-efficacy/confidence to quit has been found to predict cessation in pregnancy,^22–24^ as have general health concerns.^25^

Understanding the predictors of smoking cessation behaviors in pregnancy is valuable for informing and enhancing interventions for pregnant smokers. While demographic, or exogenous, predictors can usefully guide who might be most in need of a particular intervention, cognitive predictors are particularly important as they represent potentially modifiable targets for behavior change interventions. Pregnancy-specific harm beliefs have been found to predict both cessation^21^ and intention to quit smoking in pregnancy,^26^ and beliefs about smoking/quitting smoking can be readily targeted by interventions. Previous work has demonstrated that behavioral interventions can successfully change pregnancy-specific harm beliefs among pregnant smokers, as well as their determination and self-efficacy to quit.^27^

The smoking cessation process is conceptualized, by some behavior change theories, as occurring as part of a number of distinct stages with distinct influences.^28^ Smoking cessation research, however, has focused mainly on abstinence as its outcome of interest, with little emphasis placed on planning/initiation behaviors such as setting a quit date or making a quit attempt. Recent evidence from general adult population samples suggests that the predictors of making a quit attempt differ from the predictors of successfully quitting in those who attempt, and that this might have important implications for cessation interventions.^29,30^ In a systematic review of prospective, nonintervention studies on national/multinational adult samples,^30^ making a quit attempt was predicted consistently by intention to quit, past quit attempts and, although less frequently assessed, concerns about the effects of smoking on health. Successful abstinence in those who attempted to quit, however, was predicted consistently only by nicotine dependence. These findings were recently confirmed in a large, intervention study of general smokers^29^: motivational factors predicted making a quit attempt but not successful abstinence among those who attempted. Studies of pregnant smokers have largely failed to address the distinction between predictors of quit attempts versus quit success and we are not aware of any cross-sectional or prospective studies to investigate the predictors of quit attempts in pregnant smokers. However, these might differ from predictors among general smokers given that the motivation to quit in pregnancy is focused primarily on the baby’s health^31^ and given the fixed timeframe for quitting.

Thus, in order to better understand the process of smoking cessation in pregnancy, and thereby optimize interventions for this group, this study aimed to determine which variables were associated with attempting to stop smoking in pregnancy, and which were associated with successfully abstaining in those who attempted to stop. In particular, we focused on smoking and quitting beliefs that could be feasibly targeted by interventions, including some novel pregnancy-outcome beliefs. We investigated the relationship between these cognitive variables, together with demographic and behavioral variables, and two smoking outcomes: making a serious quit attempt during pregnancy (of at least 24 hours); and cotinine-validated abstinence, at follow-up, in those who made at least one quit attempt.

## Methods

### Design and Randomization

Pregnant smokers (*N* = 207), recruited to a trial of a self-help smoking cessation intervention,^27^ were treated as an observational cohort. Participants were randomized, at enrolment, to either receive a tailored self-help smoking cessation intervention, consisting of a tailored self-help advice leaflet and 12 weeks of tailored mobile phone text messages (“MiQuit,” *N* = 102), or to receive a non-tailored self-help leaflet only (control, *N* = 105). Texts delivered by MiQuit (0, 1 or 2 daily) include motivational messages, advice about quit attempt preparation, managing cravings and withdrawal, advice about dealing with trigger situations and preventing lapses, and information about fetal development and how smoking affects it. Full details of the intervention and control materials are available in a supplementary document to the original trial paper.^27^

Randomization was stratified by NHS Trust and baseline smoking rate (<11 vs. ≥11 cigarettes daily). Full details of the sample size calculation and randomization procedures are reported elsewhere.^27^ Participants were excluded from analyses if they experienced a miscarriage or stillbirth after enrolment.

### Participants

Participants were recruited from seven NHS Trusts in England between December 2008 and October 2009. Community midwives in each Trust were asked to invite all pregnant smokers to participate provided they met the inclusion criteria when seen at their antenatal booking appointment: aged at least 16, less than 21 weeks pregnant, smokes at least seven cigarettes weekly, has regular use of a mobile phone, and understands written English. Eligible women who were interested in participating completed a study referral form, returned by the midwife to the research team by post. Incomplete forms for those who declined to participate were also returned, with a reason if given. Women enrolled in the study by completing and returning a baseline questionnaire and consent form by post. Of 512 eligible women, 307 (60%) either declined to take part or failed to return completed forms. All 207 participants were sent a follow-up questionnaire 12 weeks after enrolment, and were contacted by text message and telephone if either questionnaire was not returned within 10 days. Participants received a £5 gift voucher for completing each of the questionnaires.

### Predictor Measures

All predictor variables were measured at baseline, via questionnaire, at approximately 13 weeks’ gestation (Table 1). Index of Multiple Deprivation (IMD) score^32,33^ was matched to participants’ home postcode as an indicator of socioeconomic deprivation. Baseline characteristics were well-balanced between groups in the original trial.^27^ Predictor variables were selected on the basis of previous research rather than preliminary analyses.

**Table 1.** Baseline Characteristics of Participants Present vs. Lost to Follow-up

	Present at follow-up (*N* = 175)		Lost to follow-up (*N* = 23)		Group difference?^a^
	Count	%	Count	%	
Intervention arm					
MiQuit	86	49.1	10	43.5	
Control	89	50.9	13	56.5	
Demographics					
IMD score^b^ (mean, *SD*)	21.00 (15.30)		22.94 (18.61)		
Age group					
Under 20	25	14.3	6	26.1	
20–29	97	55.4	11	47.8	
30–39	47	26.9	6	26.1	
≥40	6	3.4	0	0.0	
Has a partner who smokes					
Yes	117	66.9	15	65.2	
No	58	33.1	8	34.8	
Smoking/quitting behaviors					
Nicotine dependence category^c^					
Low	56	32.0	6	26.1	
Medium	74	42.3	10	43.5	
High	45	25.7	7	30.4	
Longest quit attempt prior to baseline					
Not attempted	24	13.7	7	30.4	
Less than 2 wk	52	29.7	4	17.4	
2–5 wk	30	17.1	5	21.7	
6–11 wk	17	9.7	2	8.7	
12 wk or more	52	29.7	5	21.7	
Smoked in a previous pregnancy					
Yes	93	53.4	9	39.1	
No	81	46.6	14	60.9	
Smoking/quitting beliefs					
Intention to quit					*p* = .02
Not seriously planning to quit	16	9.2	1	4.3	
Within the next 3 mo	48	27.6	3	13.0	
Within the next 30 d	62	35.6	7	30.4	
Within the next 2 wk	48	27.6	12	52.2	
Nonsmoker identity (mean, *SD*)	3.11 (1.06)		3.26 (1.18)		
Perceived social support (mean, *SD*)	3.20 (1.29)		3.65 (1.30)		
Harm to baby beliefs (mean, *SD*)	4.11 (1.07)		4.26 (1.14)		
Cutting down beliefs (mean, *SD*)	3.68 (1.11)		3.52 (1.16)		
Easier delivery beliefs (mean, *SD*)	1.72 (1.12)		1.87 (1.14)		
Determination to quit for remainder of pregnancy (mean, *SD*)	3.94 (0.99)		4.39 (0.94)		*p* = .04
Self-efficacy score (mean, *SD*)	2.68 (0.82)		2.75 (0.83)		
Interested in receiving risk information					*p* = .04
Yes	123	70.3	20	90.9	
No	52	29.7	2	9.1	

HSI = Heaviness of Smoking Index; IMD = Index of Multiple Deprivation.

^a^Tested using chi-squared test (percentages) or *t* test (means), two-tailed.

^b^IMD in England covers domains of income, employment, health, education, crime, access to services and living environment. The score refers to the proportion of people in the neighborhood who are classed as deprived.^32^

^c^Nicotine dependence was categorized using an adapted HSI,^27,34^ combining the score of two items: cigarettes per day (1–5 = score of 0, 6–10 = 1, 11–20 = 2, 21–30 = 3, >30 = 4) and time to first cigarette after waking (>2h = 0, 1–2h = 1, 31–59min = 2, ≤30min = 3). A combined score of 0–2 = low dependence, 3–4 = medium dependence, 5–7 = high dependence.

Demographic predictors modelled were Index of Multiple Deprivation score, age group, and having a partner who smokes.

Smoking and quitting behaviors modelled were nicotine dependence, quit attempt history, and having smoked in a previous pregnancy. An adapted Heaviness of Smoking Index (HSI)^27,34^ was used to class participants as low, medium or high nicotine dependence (Table 1). Quit attempt history prior to baseline was modelled as both binary (any/no quit attempt) and linear (duration of longest attempt), as both have been found to predict a quit attempt in nonpregnant smokers.^30^

Strength of intention to quit smoking was defined by if and when participants seriously planned to quit (“not seriously planning to quit,” “within the next 3 months,” “within the next 30 days,” “within the next 2 weeks”).^28^ Other smoking and quitting belief items were measured on a five-point scale from “not at all” (1) to “extremely” (5). Smoking belief items, included as individual predictors, assessed nonsmoker identity (“I can see myself as a nonsmoker”), perceived social support (“Do you have support from your family and friends to help you quit?”), and health-related evaluations of smoking in pregnancy, both positive and negative (“Smoking during pregnancy can cause serious harm to my baby,” “Cutting down would greatly reduce the health risks of smoking in pregnancy,” “Having a lower birth weight baby will make delivery easier”). Quitting belief predictors were determination to quit for the remainder of the pregnancy, and a combined self-efficacy scale (α = 0.81) score.^27^ The latter was the average of four items measuring confidence to avoid smoking: one for the remainder of the pregnancy, and three in different types of tempting situation (after a meal, with other smokers, when anxious or stressed). Participants were also asked (yes/no) if they would be interested in receiving detailed risk information about the effects of smoking in pregnancy. The theoretical basis for these measures is described in a supplementary document to the original MiQuit trial paper.^27^

### Outcome Measures

At 12-week follow-up (around 25 weeks’ gestation), participants were sent a further questionnaire to assess their current smoking status and number of quit attempts since baseline (defined as lasting at least 24 hours). Two dichotomous variables formed the outcome measures: having made at least one quit attempt in the 3-month period since baseline (self-reported), and, for those who reported at least one quit attempt, 7-day point prevalence abstinence at the 12-week follow-up point (biochemically-validated). Biochemical validation was carried out, for those self-reporting abstinence, using cotinine assessment from saliva samples sent by post, with a cut-off value of 13ng/ml.^35^ Six participants reported abstinence from smoking but reported making no quit attempt. As in other studies,^30^ these were reclassified as attempters.

### Data Analysis and Attrition

Analyses were undertaken in IBM SPSS v22, with an alpha level of *p* < .05 (two-tailed) for all statistical tests. Following similar procedures to studies included in the Vangeli et al. review,^30^ and due to relatively low attrition, we used complete case main analyses for both outcomes. Nine participants (4%) were excluded from all analyses due to miscarriage/stillbirth. A further 23 participants (11%) were lost to follow-up, leaving 175 complete cases (85%) for the quit attempt main analyses. Predictor measures were compared statistically between those lost to follow-up and those with complete outcome data (Table 1). Main analyses for the abstinence outcome were restricted to participants who made a quit attempt (*N* = 148). A further 12 participants were excluded due to not returning a viable saliva sample, leaving 136 cases with complete outcome data.

Sensitivity analyses were carried out assuming the likeliest behavioral outcomes for participants lost to follow up or validation. Sensitivity analyses for the quit attempt outcome assumed that those lost to follow-up had made a quit attempt, given that it occurred in the majority of trial participants.^27^ Sensitivity analyses for the abstinence outcome assumed that those lost to follow-up or validation had made a quit attempt but, in line with the Russell Standard for evaluating cessation interventions,^36^ were still smoking.

Binary logistic regression was used to determine significant predictors of: (1) making a quit attempt; (2) cotinine-validated abstinence in those who made a quit attempt. The same set of predictors was used in both analyses. Results are presented as odds ratios (*OR*s), with 95% confidence intervals (CIs). A univariate analysis was carried out for each predictor variable. Following collinearity checks, any predictors where *p* < .1 in univariate analyses were entered, simultaneously, into a multivariate regression model controlling for trial arm to assess their unique contribution in predicting the outcome. We also tested for interactions between trial arm and any predictors found to be significant in the multivariate models to check for any differences in the predictive relationships between the two treatment groups. A range of regression diagnostics were carried out to check for outliers and influential data points (leverage, DfBeta and Cook’s distance).

## Results

### Participant Characteristics

Participants included in analyses (*N* = 175) were 13.0 (*SD* 3.3) weeks’ gestation at baseline. All were of white ethnicity and 58% already had children. Thirty percent of participants reported smoking five or fewer cigarettes daily at baseline (compared to 4% pre-pregnancy), and 31% reported smoking more than 10 cigarettes daily (compared to 81% pre-pregnancy). Table 1 shows distributions of the predictor variables, both for participants present and for those lost to follow up. Those lost to follow-up were intending to quit smoking sooner (χ^2^(1) = 5.4, *p* = .02), had higher determination to quit for the remainder of their pregnancy (*t*(196) = 2.1, *p* = .04), and were more likely to be interested in receiving risk information (χ^2^(1) = 4.2, *p* = .04). No other measure differed between those present or missing, nor did attrition rates differ by trial arm. Over 50% of participants had smoked in a previous pregnancy. Determination to quit for the remainder of the pregnancy was high, and harm to baby beliefs received high endorsement, but only 63% intended to quit smoking within the next month.

### Outcome Event Rates

At follow-up, 148 participants (85%) had made a quit attempt in the 3 months since baseline. Of 42 participants who self-reported abstinence, 20 were biochemically confirmed as abstinent (14% of those who made a quit attempt, 11% of all participants at follow up). Ten had cotinine levels above the cut value, and were classified as smokers, and 12 did not return a valid sample, and were excluded. Smoking outcomes did not differ significantly by trial arm.

### Predictors of Making a Quit Attempt

Table 2 shows the effect of each predictor variable, in univariate and multivariate models, on each of the outcome variables. In the univariate models, five variables were significant predictors of making a quit attempt. Four of these were belief variables: intention to quit (*OR* = 2.66 [95% CI 1.61–4.37], *p* < .001); determination to quit for the remainder of the pregnancy (*OR* = 2.07 [1.40–3.08], *p* < .001); self-efficacy to quit (*OR* = 1.79 [1.05–3.05], *p* = .032); and nonsmoker identity (*OR* = 1.67 [1.08–2.59], *p* = .021). One behavioral variable, having smoked in a previous pregnancy (*OR* = 0.35 [0.14–0.87], *p* = .023), negatively predicted making a quit attempt. In addition to the significant predictors above, harm to baby beliefs, cutting down beliefs, and having a partner who smokes (*p* < .1) were entered into the multivariate model controlling for trial arm. In the multivariate model, intention to quit was the only independent predictor of making a quit attempt (*OR* = 2.41 [1.19–4.87], *p* = .014). No significant interaction was found between trial arm and intention to quit. Collinearity between predictor variables was low, and regression diagnostics were satisfactory.

**Table 2.** Predictors of Making a Quit Attempt and Predictors of Validated Abstinence in Pregnant Smokers

	Quit attempt (event rate = 148 of 175)		Validated abstinence (event rate = 20 of 136)	
	Univariate *OR* (95% CI)	Multivariate *OR* (95% CI)	Univariate *OR* (95% CI)	Multivariate *OR* (95% CI)
MiQuit arm	1.49 (0.65–3.44)	1.88 (0.70–5.10)	1.55 (0.59–4.08)	2.36 (0.81–6.93)
Demographics				
IMD score	0.99 (0.97–1.02)		1.01 (0.98–1.04)	
Age group	1.02 (0.58–1.81)		0.49 (0.24–1.00)*	0.50 (0.23–1.07)
Has a partner who smokes	2.13 (0.93–4.89)*	2.57 (0.96–6.88)	0.50 (0.19–1.34)	
Smoking/quitting behaviors				
Nicotine dependence (higher = more dependent)	0.81 (0.47–1.40)		0.27 (0.12–0.62)***	0.25 (0.10–0.60)***
Any quit attempt prior to baseline	1.32 (0.37–4.79)		0.65 (0.14–3.05)	
Duration of longest quit attempt prior to baseline	0.96 (0.73–1.28)		1.04 (0.75–1.45)	
Smoked in a previous pregnancy	0.35 (0.14–0.87)**	0.47 (0.17–1.36)	0.54 (0.20–1.48)	
Smoking/quitting beliefs				
Intention to quit (higher = stronger intention)	2.66 (1.61–4.37)****	2.41 (1.19–4.87)**	0.64 (0.38–1.08)*	0.59 (0.32–1.10)
Nonsmoker identity	1.67 (1.08–2.59)**	1.30 (0.77–2.20)	1.17 (0.74–1.84)	
Perceived social support	1.12 (0.82–1.55)		0.97 (0.66–1.42)	
Harm to baby beliefs	1.38 (0.96–1.97)*	0.76 (0.44–1.30)	1.13 (0.71–1.79)	
Cutting down beliefs	1.38 (0.96–1.98)*	1.16 (0.76–1.78)	0.92 (0.60–1.41)	
Easier delivery beliefs	1.05 (0.72–1.54)		0.94 (0.61–1.46)	
Determination to quit for remainder of pregnancy	2.07 (1.40–3.08)****	1.38 (0.71–2.65)	1.08 (0.64–1.85)	
Self-efficacy score	1.79 (1.05–3.05)**	0.81 (0.39–1.70)	1.14 (0.62–2.12)	
Interested in receiving risk information	0.67 (0.29–1.59)		1.68 (0.63–4.47)	

CI = confidence interval; IMD = Index of Multiple Deprivation; *OR* = odds ratio.

*p < .1 (entry criterion for multivariate model); **p < .05; ***p < .01; ****p < .001.

### Predictors of Validated Abstinence

In the univariate models, the only significant predictor of cotinine-validated abstinence in those who made a quit attempt was baseline nicotine dependence (*OR* = 0.27 [0.12–0.62], *p* = .002). Two further variables were included in the multivariate model controlling for trial arm: intention to quit and age group (*p* < .1). In the multivariate model, nicotine dependence was the only independent predictor of abstinence (*OR* = 0.25 [0.10–0.60], *p* = .002) and this did not interact with trial arm. Again, collinearity between predictors was low and regression diagnostics satisfactory.

### Sensitivity Analyses

Sensitivity analyses for the quit attempt outcome, where participants lost to follow-up (*N* = 23) were included and assumed to have made a quit attempt, resulted in the exclusion of cutting down beliefs from the multivariate model (*p* > .1), but had otherwise negligible effects on either the univariate or multivariate model results. Sensitivity analyses for the abstinence outcome, where those lost to follow-up (*N* = 23) or to validation (*N* = 12) were assumed to have made a quit attempt but still be smoking, resulted in intention to quit becoming a significant negative predictor of abstinence in both the univariate (*OR* = 0.59 [0.35–0.99], *p* = .044) and multivariate models (*OR* = 0.55 [0.31–1.00], *p* = .048).

## Discussion

### Summary of Main Results

This is the first study, to our knowledge, to separate the predictors of quit attempts in pregnancy from the predictors of achieving abstinence in those who make a quit attempt. The findings indicate that, among pregnant smokers, different factors predict each of these behaviors. Cognitive factors, particularly those relating to motivation to quit, were predictive of attempts to quit smoking, whereas only nicotine dependence predicted abstinence in those who made at least one quit attempt. This corresponds with previous research on general adult smoker populations,^29,30^ which concludes that motivational factors are important to initiating a quit attempt, but dependence is the major obstacle to maintaining abstinence in those who attempt to quit.

Intention and determination to quit emerged as strong univariate predictors of making a quit attempt in our cohort. These variables were high in our cohort, as might be expected, with only a minority not seriously planning to quit (9%), and a high proportion (85%) made a quit attempt in comparison to rates of 20% to 60% reported in studies of nonpregnant smokers^29,30^ As with nonpregnant smokers, however, and despite their high determination and intention to quit, these variables did not predict successful abstinence among attempters. Our study therefore indicates that motivation is likely to be important in initiating a quit attempt in pregnancy, but not in maintaining abstinence. One reason postulated for why motivation does not predict abstinence among general smokers is that those who are highly motivated to quit may also be more nicotine dependent,^37^ but this was not supported by our data as, during assessments of collinearity, nicotine dependence and motivation were not related. An alternative suggestion for explaining why motivation does not predict abstinence is that high motivation could actually hinder abstinence; those with high motivation to quit believe that this will be sufficient for success, making them less likely to use cessation support such as Nicotine Replacement Therapy.^37^ In support of a negative influence of motivation on cessation success, it is noteworthy that our participants lost to follow-up had higher intention to quit, with 52% intending to quit within the next 2 weeks. When included in analyses under the standard assumption that missing intervention participants are still smoking (which recent evidence suggests is likely to be valid),^38^ intention to quit negatively predicted abstinence, although more weakly than dependence. Motivational factors may therefore be insufficient, or even potentially counter-productive, to promote abstinence in the face of cravings and other nicotine withdrawal symptoms likely to be experienced by many women in the early stages of a quit attempt.

Having smoked in a previous pregnancy was a significant negative predictor of making a quit attempt in this cohort. Women who had smoked in a previous pregnancy (the majority of those who were not primiparous) had around one-third the odds of attempting to quit than women who had not. Qualitative research has highlighted that smoking in a previous pregnancy, yet delivering a seemingly-healthy baby, can pose a significant barrier to quitting because it allows women to challenge the health risks of smoking to the fetus.^39,40^ While pregnancy-specific harm beliefs were largely endorsed by the women in our sample, research shows that harm beliefs can be downplayed in pregnant smokers under certain circumstances, or the risks seen as less personal.^39,40^ A lack of understanding of the exact mechanism of damage to the fetus has also been highlighted as a barrier to quitting among pregnant smokers, despite their acceptance of the risks.^40^ Challenging pregnant smokers’ misconceptions about harms may therefore be beneficial, particularly to women who have smoked in a previous pregnancy.

Nicotine dependence emerged as the only significant predictor of cessation success among pregnant smokers who made a quit attempt. It was unrelated to the probability of making a quit attempt in our cohort, however, suggesting that even highly dependent smokers can be facilitated to initiate a quit attempt in pregnancy given the motivation to do so. The importance of nicotine dependence to quit attempt failure is very well documented, both in pregnancy^10^ and outside of pregnancy.^29,30^ Most pregnant smokers try to quit on their own, without professional support,^41^ and it seems likely that the more nicotine dependent will require extra help to succeed once they begin a quit attempt.

### Implications for Smoking Cessation Interventions

The variables identified in this study as predictive of making a quit attempt in pregnancy are largely cognitive in nature, and thus useful targets for interventions. Our results indicate that interventions should tackle the smoking cessation process in pregnancy as, at least, a two-stage process. Regardless of nicotine dependence level, the quitting motivation of pregnant women who continue to smoke should be targeted with the aim of increasing their likelihood of making a quit attempt. Our results, and those of previous research,^27^ lead us to expect that targeting the smoking and quitting beliefs of pregnant smokers could prompt a higher proportion to initiate a quit attempt by increasing their motivation and self-efficacy to quit. Specifically challenging the risk perceptions of women who have smoked in a previous pregnancy also appears warranted.

Once women are engaged in quitting, however, the aim of an intervention becomes the maintenance of abstinence, and our results indicate that motivational factors are less important here than level of nicotine dependence. Interventions supporting pregnant women after initiation of a quit attempt, therefore, may do better to focus their efforts not on quitting motivation, but on the monitoring and management of withdrawal symptoms, and on avoiding and coping with relapse situations. In more dependent smokers, interventions that include the management of nicotine withdrawal, for example, Nicotine Replacement Therapy, are likely to be most important.

A recent study in an antenatal setting illustrates how smoking cessation rates can be improved by motivating pregnant smokers to make a quit attempt that utilizes the support of NHS Stop Smoking Services,^42^ which includes pharmacotherapy to manage nicotine withdrawal. Stop Smoking Services support is accessed by a minority of pregnant smokers, despite a reported short-term success rate of 45% among those who do.^41^ Carbon monoxide screening of pregnant women at their antenatal booking appointment, and an opt-out referral system, more than doubled the numbers who then engaged with Stop Smoking Services by setting a quit date, compared to a previous opt-in system, and a qualitative study of the same women found they viewed the carbon monoxide screening as valuable in increasing their quitting motivation.^43^ Over 75% of pregnant smokers who set a quit date with Stop Smoking Services via the opt-out system reported abstinence 4 weeks later, similar to the previous opt-in proportion,^42^ suggesting that motivating more pregnant smokers to make a supported quit attempt would be worthwhile in terms of cessation rates.

### Strengths and Weaknesses

This study is the first to investigate the predictors of quit attempts in pregnancy separately from the predictors of abstinence among attempters. It is also the first to investigate a broad range of cognitive, behavioral and demographic predictors of quit attempts in pregnancy, including some novel, pregnancy-outcome beliefs. We used a biochemically-validated abstinence measure and achieved low attrition rates.

Limitations of this study include the relatively small sample size and, consequently, power to detect only relatively strong predictive relationships, particularly for the validated abstinence outcome. Although our results are in agreement with those from much larger studies on general smoker populations, it is possible that further independent predictors would have emerged given a larger sample, such as having a partner who smokes on the quit attempt outcome, or lower age, as well as lower quit intention, on successful abstinence. Biochemically-validated abstinence rates were low but we favored these for their high validity compared to self-report, and we have identified potential predictors of validated abstinence among pregnant smokers who make a quit attempt for future investigation. It is also possible that we failed to include unknown, important predictor variables, but we aimed to include all those identified as consistent predictors in previous research, or a similar proxy, as well as novel, pregnancy-specific beliefs. Given that our cohort was from an intervention study where 60% of those eligible declined to take part, baseline motivation to quit might have been higher than among pregnant smokers in general. However, the proportion who planned to quit within the next month (63%) was similar to that found among pregnant smokers surveyed in England shortly after their first antenatal booking visit (70%),^12^ so our sample is likely to be fairly representative of the population in terms of quit motivation. We also used only a single-point outcome measure, taken at around 6 months’ gestation, and it is not known whether participants relapsed after giving birth. However, it is likely that different factors, again, predict maintaining abstinence after delivery.^16^

## Conclusions

In pregnant smokers, the predictors of making a quit attempt differ from the predictors of maintaining abstinence in those who try to quit. As in general smokers, cognitive variables, notably intention and determination to quit, predict making a quit attempt during pregnancy but do not predict successful abstinence among attempters, where nicotine dependence dominates. Interventions could utilize these findings by targeting motivation to quit while pregnant women are continuing to smoke, but focusing on coping with withdrawal once they are engaged in quitting.

## Funding

This work was supported by Cancer Research UK (CR-UK) (grant number C1345/A5809), and by a Society for the Study of Addiction (SSA) Fellowship.

## Declaration of Interests

*None declared.*
